# Transcriptomic profiles reveal the characteristics of oocytes and cumulus cells at GV, MI, and MII in follicles before ovulation

**DOI:** 10.1186/s13048-023-01291-2

**Published:** 2023-11-22

**Authors:** Yena Hu, Ran Zhang, Shuoping Zhang, Yaxing Ji, Qinwei Zhou, Lizhi Leng, Fei Meng, Fei Gong, Guangxiu Lu, Ge Lin, Liang Hu

**Affiliations:** 1 Institute of Reproductive and Stems Cell Engineering, NHC Key Laboratory of Human Stem Cell and Reproductive Engineering, School of Basic Medical Science, Central South University, Xiangya Road 88#, Changsha, 410008 Hunan China; 2 Clinical Research Center for Reproduction and Genetics in Hunan Province, Reproductive and Genetic Hospital of CITIC-XIANGYA, Changsha, 410013 Hunan China; 3 Hunan International Scientific and Technological Cooperation Base of Development and Carcinogenesis, Changsha, 410013 Hunan China; 4 National Engineering and Research Center of Human Stem Cells, Changsha, 410013 Hunan China; 5 Hunan Normal University School of Medicine, ChangshaHunan, 410013 China

**Keywords:** Transcriptome, Cumulus cell, Oocyte, Oocyte maturation

## Abstract

**Background:**

The oocyte and its surrounding cumulus cells (CCs) exist as an inseparable entity. The maturation of the oocyte relies on communication between the oocyte and the surrounding CCs. However, oocyte evaluation is primarily based on morphological parameters currently, which offer limited insight into the quality and competence of the oocyte. Here, we conducted transcriptomic profiling of oocytes and their CCs from 47 patients undergoing preimplantation genetic testing for aneuploidy (PGT-A). We aimed to investigate the molecular events occurring between oocytes and CCs at different stages of oocyte maturation (germinal vesicle [GV], metaphase I [MI], and metaphase II [MII]). Our goal is to provide new insights into in vitro oocyte maturation (IVM).

**Results:**

Our findings indicate that oocyte maturation is a complex and dynamic process and that MI oocytes can be further classified into two distinct subtypes: GV-like-MI oocytes and MII-like-MI oocytes. Human oocytes and cumulus cells at three different stages of maturation were analyzed using RNA-seq, which revealed unique transcriptional machinery, stage-specific genes and pathways, and transcription factor networks that displayed developmental stage-specific expression patterns. We have also identified that both lipid and cholesterol metabolism in cumulus cells is active during the late stage of oocyte maturation. Lipids may serve as a more efficient energy source for oocytes and even embryogenesis.

**Conclusions:**

Overall, our study provides a relatively comprehensive overview of the transcriptional characteristics and potential interactions between human oocytes and cumulus cells at various stages of maturation before ovulation. This study may offer novel perspectives on IVM and provide a reliable reference data set for understanding the transcriptional regulation of follicular maturation.

**Supplementary Information:**

The online version contains supplementary material available at 10.1186/s13048-023-01291-2.

## Background

For human reproduction, a fully developed oocyte is essential. The maturation of the oocyte, which is surrounded by cumulus granulosa cells (CCs), is a complicated process that relies on the oocyte itself and the communication between the oocyte and the surrounding CCs [1, 2].

As follicles develop, granulosa cells differentiate into two types: mural granulosa cells and cumulus cells. Mural granulosa cells are mainly responsible for hormone production, while CCs surround the oocytes and support their growth and maturation [3]. Oocytes and CCs progress together through bidirectional communication [1, 4], which occurs via gap junctions through the zona pellucida (TZP) and paracrine signaling. Some metabolic substrates and small molecules are exchanged between oocytes and CCs via gap junctions and paracrine pathways. Overall, oocytes secrete various factors that regulate the proliferation, differentiation, and apoptosis of CCs [5–8]. In turn, CCs provide oocytes with the necessary energy source for their growth and development [9–13].

Currently, oocyte evaluation is primarily based on morphological parameters, which provide little insight into oocyte quality and competence. Additionally, current procedures for in vitro oocyte maturation (IVM) are inadequate and do not give suitable options in cases where oocyte maturation disorders result in the retrieval of many immature oocytes despite properly monitored ovarian stimulation. As a result, molecular studies of the processes involved in nuclear and cytoplasmic maturation, as well as potential diseases, in COCs may provide new insights into IVM.

This study aimed to explore the transcriptomes of the final three stages of oocytes and CCs before ovulation, to create a reliable reference dataset that can aid in comprehending the transcriptional regulation of follicular maturation.

## Results

### Global transcriptome profiling of human oocytes and CCs

**Fig. 1:**
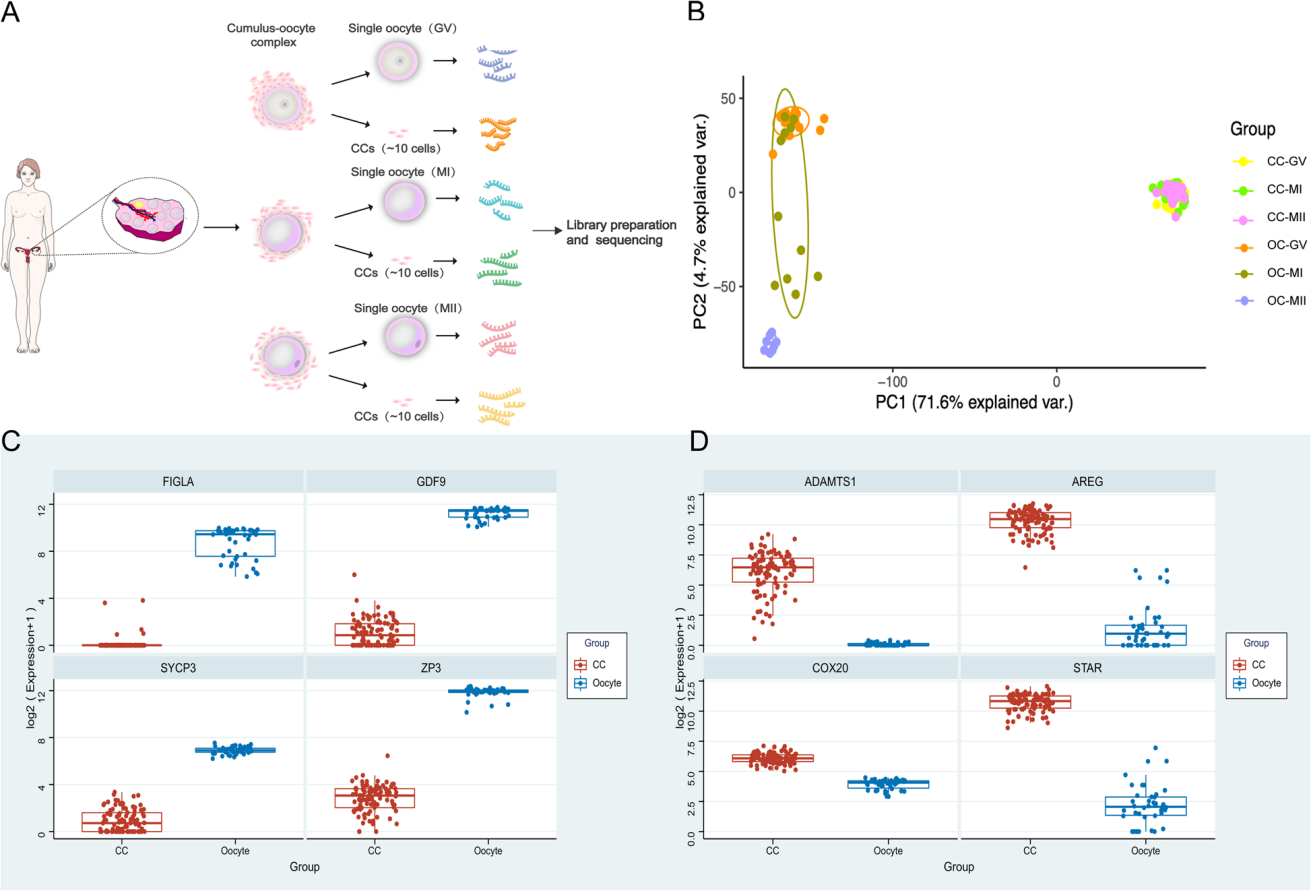
The global transcriptome patterns of human oocytes and CCs at the different maturation stages. **A** Schematic illustration of the study workflow. germinal vesicle (GV, oocyte maturation arrested in the prophase of meiosis), metaphase I (MI, first meiotic metaphase), and metaphase II (MII, second meiotic metaphase). **B** Principal component analysis (PCA) of all samples shows significant separation between oocytes and CCs along PC1 by oocyte maturity but no apparent clustering within the CCs group showed by PC2. **C** Expression patterns of oocyte marker genes and **D** CCs maker genes in all samples; The CCs are shown in red, and the oocyte is shown in blue

Upon obtaining ethical approval, we collected a total of 139 samples from 47 patients undergoing preimplantation genetic testing (PGT). The patients had an average age of 35.1 years (Supplementary Tables 1 and 2) for RNA sequencing (Fig. 1A), including 97 CCs (GV: 35; MI: 30; MII: 32) and 42 oocytes (GV: 22; MI: 11; MII: 9). The quality of the sequencing results from all samples met the requirements for further analysis.

The first principal component (PC1) was found to be mainly influenced by differences between oocytes and CCs. PC2, on the other hand, reflected differences among the CC cohorts, amounting to 71.6% and 4.7%, respectively (Fig. 1B). This suggests that oocytes and CCs can be clustered into two distinct populations that are not dependent on the maturation stage. The average amount of raw data per sample was about 3 GB. Low expression data and individual-specific expression data were filtered out to obtain significantly differentially expressed genes (DEGs) with less bias. To identify transcripts that are specifically enriched at each maturation stage, we considered genes with a log2-fold change ≥ 2 and a *p*‐value < 0.05 as DEGs. We identified a total of 7460 DEGs between the two cell populations, among which 3982 were significantly highly expressed in oocytes, and 3478 in CCs (Supplementary Fig. 1). Next, the expression levels of known cell type-specific markers for oocytes (*FIGLA*, *GDG9*, *SYCP3*, *ZP3*) [3] and CCs (*ADAMTS1*, *AREG*, *COX20*, *STAR*) [5] were analyzed. The results showed that the marker genes for oocytes were significantly highly expressed in oocytes, while the marker genes for CCs were significantly highly expressed in CCs (Fig. 1C, D).

### Transcriptional Characteristics of Oocytes during Maturation

**Fig. 2:**
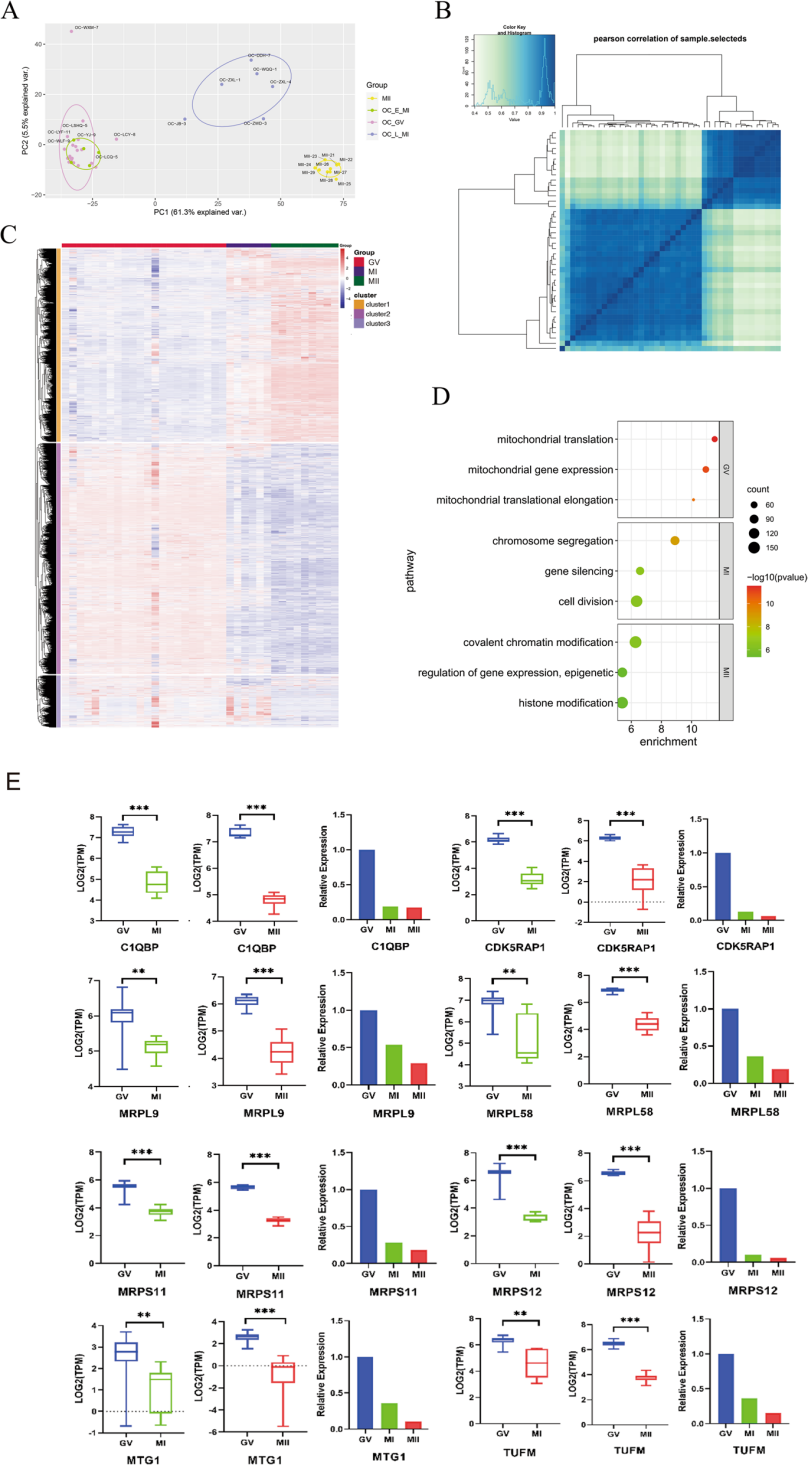
Unique transcriptome analysis of oocytes. **A** PCA of the transcriptome of RNA-seq data from oocytes at different stages of maturation. oocytes were clustered into three subpopulations corresponding to the maturation stages. **B** Pearson correlation analysis between samples of oocytes. **C** Heatmap of differential genes in 3 stages of oocytes. **D** Enrichment results of transcriptionally active genes in GV, MI, MII 3 periods. **E** Expression of genes related to mitochondrial translation in GV, MI, and MII stages (gene expression levels in the GV stage were normalized)

Principal component analysis (PCA) showed that all GV oocytes and MII oocytes were well separated, while MI oocytes were clustered into two groups. Five of the MI oocytes were clustered with GV oocytes, while the remaining six were in the middle of the GV and MII oocytes population (Fig. 2A). This was further confirmed by Pearson correlation, which showed that the five MI oocytes were highly correlated with GV oocytes, and the remaining six were positioned between GV oocytes and MII oocytes. This suggests that MI oocytes can be classified into two subgroups: GV-like MI oocytes and MII-like MI oocytes (Fig. 2B). To represent the transcriptional profile of MI oocytes more accurately, we used the six MII-like-MI oocytes as the sample for subsequent analysis. We analyzed DEGs that were highly expressed in oocytes at different stages. Genes are organized into three clusters. Custer1 shows high expression in the MII cohort, while cluster2 is expressed in the GV cohort. Cluster 3 appears to be a gene set that is intermediate between the two (Fig. 2C). Genes that are highly expressed in GV oocytes are annotated to mitochondrial translation, mitochondrial gene expression, and mitochondrial translation elongation. Genes that are upregulated in MI populations are associated with processes such as chromosome segregation, gene silencing, and cell division. Genes that are upregulated in MII populations are involved in covalent chromatin modification, regulation of gene expression, epigenetics, and histone modification (Fig. 2D). Given the significant role of the mitochondrial translation pathway, we conducted a screening for genes that are enriched in this pathway to observe their expression at three different stages. We then normalized the expression in the GV stage. The results showed that these genes were highly expressed during the GV stage, which decreased during the MI stage, and had the lowest expression during the MII stage (Fig. 2E). This indicates that mitochondria are active during the GV stage, and the GV stage oocytes require a large amount of energy to support their completion of meiosis [14]. As oocytes mature to the MII stage, mitochondrial function and energy substrate utilization gradually decline. Some of the unused energy substrates may be stored [15].

### Transcription factor regulatory networks in the oocytes

**Fig. 3:**
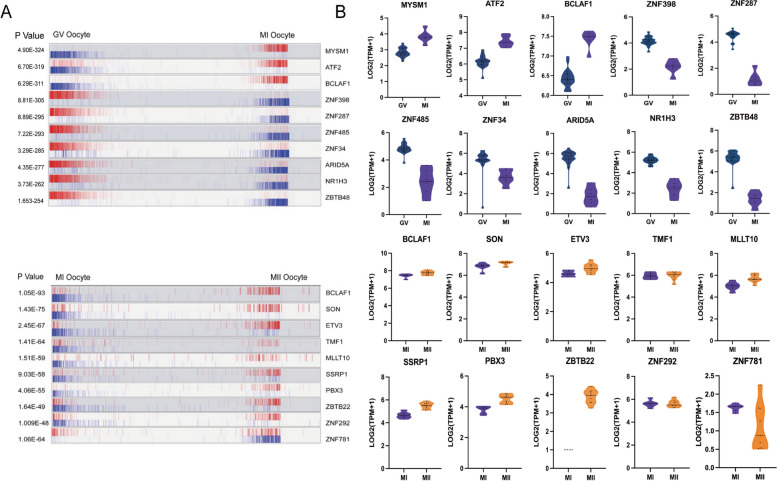
Inferred Key Transcriptional Factors in Oocytes at Each Stage-to-Stage Transition of Folliculogenesis **A** FET plots of targets for each candidate master regulator. The red vertical bar represents the activated targets; the blue vertical bar represents the repressed targets. On the x-axis, genes are rank sorted according to the significance of differential expression between the two developmental stages. The candidate master regulators are displayed on the right. The corresponding p values of these master regulators indicating the significance of enrichment are displayed on the left. **B** Violin plots show the relative expression levels (log2 [TPM + 1]) of each master regulator in two consecutive stages

To investigate the key regulators and establish a transcriptional regulatory network during the maturation of human preovulatory oocytes, we utilized the ARACNe method to analyze all 1665 known transcription factors (TFs) from the Human Transcription Factor Database (TFDB v3.0) [16, 17]. We observed the upregulation of *MYSM1*, *ATF2*, and *BCLAF1* in MI oocytes, which indicates that these TFs may play a critical role in the transition from the GV to the MI stage. *BCLAF1*, *SON*, *ETV3*, *TMF1*, *MLLT10*, *SSRP1*, *PBX3*, *ZBTB22*, and *ZNF292* were found to be highly expressed in the oocytes from MII oocytes compared to those from MI oocytes (Fig. 3), implying that they are likely the regulators of the MI-to-MII stage transition. Interestingly, we found that *BCLAF1* was upregulated in oocytes during both the GV-to-MI and MI-to-MII transitions. These may suggest a potential role for *BCLAF1* in regulating transcription networks during human oocyte maturation before ovulation. It is worth noting that the transcriptional regulatory network between the three stages of CCs cannot be constructed due to the limited number of differentially expressed genes. This may require additional investigation.

### Transcriptional characteristics of CCs during oocyte maturation

When CC samples were analyzed using PCA, the results showed that all CC samples clustered loosely according to the development period (Supplementary Fig. 2A). Heatmaps of all genes in the CCs showed no transcripts that were specifically expressed during different stages of follicle maturation (Supplementary Fig. 2B).

**Fig. 4:**
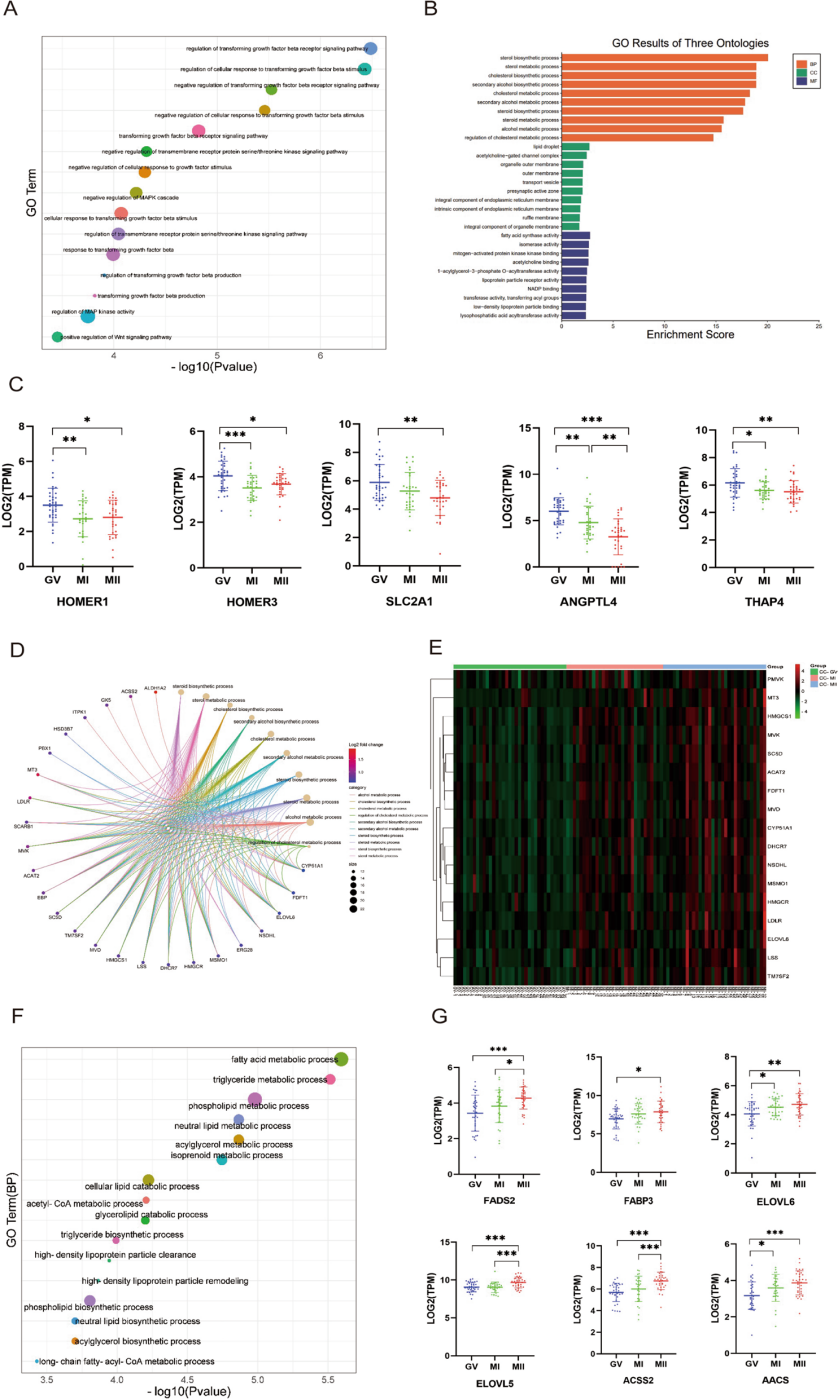
Overview of CC’s transcriptome profile. **A** GO enrichment of differentially up-regulated genes in GV CC. The sizes of the points indicate the number of genes enriched in the pathway. The X-axis indicates significance. **B** GO results of three ontologies of CCs at the MII stage. **C** Differential gene expression patterns of transcripts related to amino acid metabolism and sugar metabolism in three periods. GV, blue; MI, green; MII, red. **D** The biological process in the GO results of the differentially highly expressed genes in the MII CC, and the Enrichment profile of these pathways. **E** Heat map of the expression of genes involved in de novo cholesterol synthesis at different maturation stages. **F** The Biological pathways of genes enriched to molecular function of CCs in the MII stage. The sizes of the points indicate the number of genes enriched in the pathway. The X-axis indicates significance. **G** Expression changes of key genes in fatty acid anabolism pathway at three different periods. *FADS2* (Fatty Acid Desaturase 2). *FABP3* (Fatty Acid Binding Protein 3). *ELOVL6* (ELOVL Fatty Acid Elongase 6). *ELOVL5* (ELOVL Fatty Acid Elongase 5). *ACSS2* (Acyl-CoA Synthetase Short Chain Family Member 2). *AACS* (Acetoacetyl-CoA Synthetase). GV, blue; MI, green; MII, red

However, upon comparing GV CCs and MII CCs, there were more DEGs (log2-fold change > 0.5 and *p*‐value < 0.05) enriched in functional pathways. The comparative analysis of DEGs abundance between CCs at the GV stage and CCs at the MII stage showed that 148 transcripts were significantly downregulated in the CCs at the MII stage. KEGG and GO enrichment analyses showed that the DEGs in GV CCs were mainly related to negative regulation of plasminogen activation, regulation of the MAPK signaling pathway cascade, regulation of protease activity, regulation of the transcriptional growth factor β (TGF-β) receptor signaling pathway cascade, transmembrane receptor protein serine/threonine, tyrosine kinase signaling, and the classical Wnt signaling pathways (positive regulation). These genes are responsible for cell–cell interactions [18–20] Fig. 4A).

In addition, although we did not find any association between glucose metabolism in the annotated signaling pathways enriched in the DEGs, we did find that two genes associated with glucose transport were highly expressed in GV CCs. Solute Carrier Family 2 Member 1 (SLC2A1) is responsible for the facilitated diffusion of glucose and interacts with Angiopoietin-like 4 (ANGPTL4), which is also highly expressed in the GV phase. Furthermore, we discovered that among the few DEGs in GV CCs vs. MII CCs, THAP4, HOMER1, and HOMER3, which are related to glucose homeostasis, were significantly highly expressed in the GV CC cohort. This suggests that these genes are involved in glucose metabolism during the early stages of follicular development (Fig. 4C). Similarly, some terms concerning amino acid metabolism were found to be enriched in DEGs in the GV phase. The transcriptomes of CCs revealed the contributions of amino acid and glucose metabolism contribute to follicular metabolism during the early stage of oocyte maturation.

To further investigate the roles of transcripts of CCs in the later stages of oocyte maturation, we also conducted an enrichment analysis of 117 genes upregulated in MII CCs. The GO and KEGG analyses revealed many pathways related to lipid metabolism, including cholesterol anabolism and FA metabolism (Fig. 4B, D). MII CC cohort genes that were differentially up-regulated were annotated to cholesterol biosynthesis and the steroid and alcohol metabolic pathways, indicating that sterol metabolism of CC is a significant factor in oocyte development which is consistent with previous animal studies [21–25]. Subsequently, we conducted a cluster heatmap analysis of 17 transcripts involved in cholesterol metabolism. We observed a significant increase in the expression levels of these genes as oocyte development progressed (Fig. 4E). We investigated the expression profiles of several key genes involved in the cholesterol metabolism pathway during the GV, MI, and MII phases. These genes exhibited low transcriptional activity during the GV stage but were upregulated in MI CCs, eventually reaching a high level of expression in MII CCs (Supplementary Fig. 2C).

The GO analysis also showed that MII CCs exhibited enrichment in transcripts associated with fatty acid (FA) metabolism (Fig. 4B). To obtain a comprehensive understanding of changes in FA metabolism, we examined biological pathway terms related not only to FA metabolism but also to catabolism. Additionally, we analyzed terms related to triglyceride (TG) and acyl-CoA metabolism (Fig. 4F). Next, we analyzed the expression levels of some FA synthetase-encoding genes, including *FADS2*, *FABP3*, *ELOVL5*, *ELOVL6*, *ACSS2*, and *AACS*, at the GV, MI, and MII stages. The expression of all these genes gradually increased as the developmental stage progressed and reached its peak in the preovulatory follicles (Fig. 4G). The qPCR results of several genes confirmed our findings (Supplementary Fig. 3). We observed an increase in FA synthesis in the follicles as they matured, indicating that FA accumulation peaks before ovulation.

### The energy source of oocytes during oocyte maturation

**Fig. 5:**
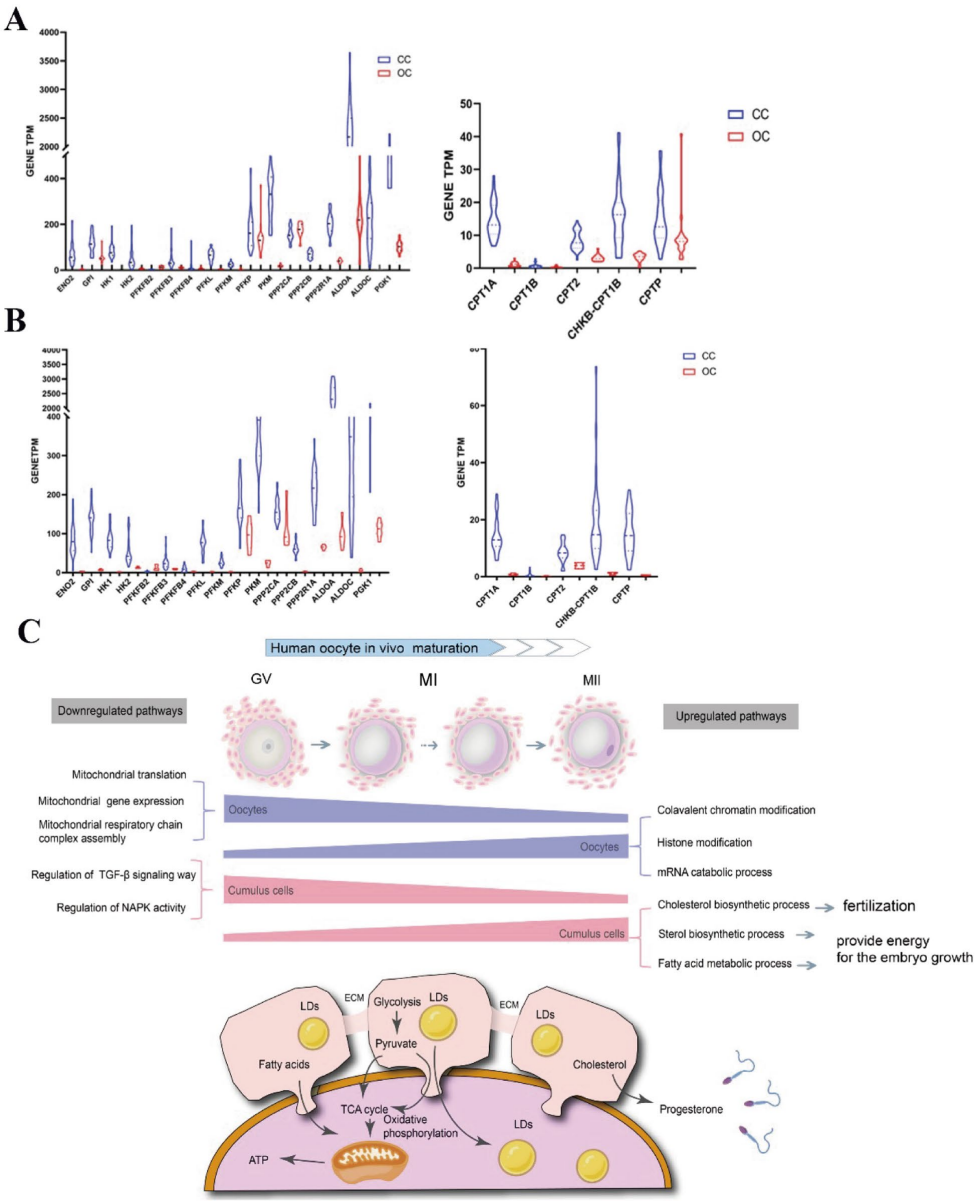
Crosstalk in energy metabolism between CCs and oocytes at different maturation stages. **A** Expression of genes associated with glycolysis (left) and fatty acid β -oxidation (right) pathways in oocytes (red) and CCs (blue) at the GV stage. **B** Expression of genes associated with glycolysis (left) and fatty acid β -oxidation (right) pathways in oocytes (red) and CCs (blue) at the MI stage. **C** Our hypothetical pattern of energy metabolism interaction between oocytes and corresponding surrounding CCs

To determine the energy source utilized during early oocyte maturation, we analyzed the expression of genes involved in two main biological energy production processes: glycolysis, and FA β-oxidation, in oocytes and CCs. We discovered that the abundance of genes associated with the glycolysis pathway was significantly higher than that of genes associated with the FA β-oxidation pathway in the GV and MI stages. We also found that the abundance of genes associated with the glycolysis pathway in CCs is much higher than in oocytes (Fig. 5A,B), implying that the primary energy source of oocyte maturation is provided by CCs’ glycolysis.

## Discussion

Our study offers valuable insights into the transcriptome and transcriptional regulatory landscape of oocytes and cumulus cells during the last stages of human follicular maturation before ovulation. The stage-specific genes, pathways, and transcriptional regulatory networks identified in oocytes and CCs may provide valuable clues for future functional studies. One study has explored the transcriptome landscape during follicle maturation [26], specific marker gene sets were identified for oocytes and cumulus cells at specific stages of follicular development (primordial, primary, secondary, antral, preovulatory). However, they did not subdivide oocytes into different maturation stages (GV stage, MI stage, MII stage). Another study showed the transcriptome of cumulus granulosa cells during the last three stages of preovulation follicle maturation [27]. Our results support the conclusions of this paper and provide new insights into the possible interactions between oocytes and cumulus cells from a transcriptional perspective. In this study, we only evaluated the transcriptional profile of CCs closely surrounding the oocyte. These CCs play a key role in the final stages of oocyte maturation by directly contacting the oocyte through TZP and delivering metabolic substrates. However, the transcriptional profile of mural granulosa cells during the final stages of follicle maturation has not been fully explored [28].

We characterized the transcriptional profiles of oocytes and CCs at the final stage of follicular maturation. The data obtained can be used to assess oocyte maturation and reproductive potential. Moreover, we found that MI oocytes can be classified into two groups based on their resemblance to either GV or MII oocytes. This is likely due to asynchronous maturation of the cytoplasm and nucleus, with the former maturing more slowly than the latter [29]. Indeed, it is usually more difficult to define and detect cytoplasmic maturation in oocytes than nuclear maturation [30]. The latter is accompanied by various cytoplasmic changes, such as the rearrangement of intracytoplasmic organelles (the endoplasmic reticulum, mitochondria, cortical granules, etc.), the compositional changes, and increased rates of transcription and translation in the cytoplasm [31]. Our findings suggest that the nucleus of MI oocytes has reached maturity when observed under the microscope during IVM. However, it may be necessary to allow for sufficient time to promote cytoplasmic maturation. More research is needed to determine the stage of cytoplasmic maturity and improve IVM outcomes in assisted reproductive technology (ART).

This study also found that as follicles mature, cholesterol synthesis by CCs increases. It has been suggested that some of the cholesterol present in the follicle may be utilized to synthesize progesterone. Additionally, as the follicle matures, there may be chemical attractants surrounding the oocyte that aid in attracting sperm for successful fertilization. These attractants may consist of CC-synthesized cholesterol-based progesterone [32]. The active metabolism of lipids in CCs involves not only alcohols like cholesterol but also FAs. Our data show that FA metabolism was active during the MII stage, with anabolism being more pronounced than catabolism. This indicates that CCs are accumulating FAs during oocyte maturation. FAs are esterified and then integrated into triglycerides (TGs) during the TG biosynthesis process. TGs, neutral lipids, and cholesterol esters are the main components of lipid droplets (LDs), which exist as energy storage reservoirs in the cytoplasm [33, 34]. The transcriptome analysis in this study revealed an increase in the expression of genes related to the “neutral lipid metabolic process”, “TG metabolic process”, and “cholesterol esterification”, indicating active LD formation. In addition, highly expression of the scavenger receptor (*SCARB1*) [35] that uptakes cholesterol esters from high-density lipoprotein (HDL) was also observed in the MII CC cohort, confirming the activation of cholesterol esterification. Other genes, such as *SCARB1*, *LPIN1*, *PNPLA3*, *LDLR*, *MBOAT7*, *FDFT1*, *HMGCS1*, *PCYT2*, *MVD*, *LIPG*, *MVK*, and *FABP3*, play a role in the biological metabolism and storage of TGs. When oocytes are exposed to a high concentration of free FAs, LDs quickly accumulate in the COC [36, 37], which prevents excessive free FAs in the follicular fluid from causing lipotoxic damage to the oocytes [38]. Consequently, it is important to maintain the functional activity involved in the formation of LDs. These LDs may be transported to the oocyte through interstitial junctions, and they served as a source of energy for subsequent embryonic development.

Unesterified FAs and cholesterol serve as the structural foundation for the synthesis of steroid hormones, which play a crucial role in female reproduction, particularly in promoting COC maturation in the preovulation period [39, 40]. In our study, we found that the expression of *FABP3*, a cytoplasmic FA transporter, suggests that certain free FAs in the follicular fluid can be transported to oocytes via gap junctions. LDLR and SCARB1 are lipoprotein receptors that play a crucial role in the active transport of lipids. Above all, the analysis of transcripts involved in adipogenesis, lipolysis, FA oxidation, and FA transport revealed active lipid metabolism in the peri-oocyte CCs.

Generally, mitochondrial respiratory chain activity is associated with the production of energy. The downregulation of transcripts associated with energy synthesis indicates a decrease in the demand for ATP. Before ovulation, the GV stage is the primary stage during which the oocyte requires energy. This finding is similar to what has been reported in mice [41, 42], suggesting that ATP consumption is mainly used to block meiosis at the GV stage. In addition, the *SLC2A1* and *ANGPTL4* genes were found to be upregulated in GV CCs, thus confirming this discovery. SLC2A1 is involved in regulating glucose homeostasis, glucose metabolism, and insulin sensitivity by interacting with ANGPTL4 [43–45]. To determine the source of energy, we analyzed the overall level of energy metabolism and observed that transcripts related to glycolysis and β-oxidation were expressed at lower levels in GV oocytes compared to GV CCs. Additionally, the expression levels of transcripts related to glycolysis were higher than those of transcripts related to oxidation (Fig. 5A). The energy required by oocytes at the GV stage is primarily derived from glycolysis in CCs [46], which can fulfill their immediate energy requirements. The continuous high level of glycolysis in MI CCs may be utilized to expand COCs and facilitate oocyte meiosis, thereby preventing the occurrence of aneuploidy. Meanwhile, their high energy levels may provide CCs with a significant antioxidant capacity, similar to that of oocytes, which can enhance their defense against oxidative stress. This has been reported in mice [47].

To further investigate the potential impact of age on oocyte and CC transcriptomic profiling, we categorized oocytes (GV, MI) and CCs from different stages (GV, MI, MII) into two groups: those aged ≤ 30 and those aged ≥ 40 years (Supplementary Table 3). Since none of the patients who donated MII oocytes were aged ≥ 40 years, we were unable to categorize and further analyze them by age.

We found that there were only a few DEGs identified between each pair of groups of all three stages of CC and GV oocytes. However, about 500 DEGs were identified between the two groups in MI oocytes. Genes that are upregulated in aged ≥ 40 years MI oocyte populations are involved in various biological processes, including histone modification, regulation of phosphatidylcholine metabolic process, and chromosome segregation (Supplementary Fig. 4). The results indicate that the age of patients may affect the transcriptomic profile of MI oocytes. But it has little effect on the transcriptomic profile of GV oocytes and CCs. The impact of age on the transcriptome profile of MII oocytes should be further studied in the future.

Our study may provide new insights into the clinical assessment of oocyte maturation stages and provide references for determining cytoplasmic maturation stages. We believe that future research will focus on determining the stage of cytoplasmic maturation using additional methods, such as metabolomics and translation omics. Additionally, researchers will work on developing methods to enhance oocyte cytoplasmic maturation, to improve outcomes for IVM.

## Conclusion

Our study provides a relatively comprehensive overview of the transcriptional features and potential interactions between human oocytes and CCs at various stages of maturation before ovulation. CCs are responsible for the accumulation of lipids and fatty acids during the late stages of oocyte maturation. This ensures that the oocyte is prepared for fertilization and that there is sufficient energy available for subsequent embryonic development. This study may offer novel perspectives on IVM and provide a reliable reference data set for understanding the transcriptional regulation of follicular maturation.

## Methods

### Sample collection

A total of 42 oocytes and 97 corresponding CCs were collected voluntarily after obtaining written informed consent from 47 donor couples, who were undergoing Preimplantation Genetic Testing for Aneuploidies **(**PGT-A**)** at the Reproductive & Genetic Hospital of CITIC-XIANGYA, using standard antagonist protocols. Patients with a history of chromosomal abnormalities or a family history of genetic abnormalities, or who have been found to have abnormal karyotypes were excluded. Couples who donated oocytes were informed that the donation posed a potential risk to their fertility success for that cycle. The MII oocytes were donated by couples who had > 20 oocytes derived from the same IVF or ICSI cycle. No financial benefit was involved in the donation process. This study was approved by the Ethics Committee of CITIC XIANG-YA Reproductive and Genetic Hospital (NO: LL-SC-2019–005).

Each cumulus-oocyte complex (COC) was placed in a single droplet, digested with 80 IU/ML hyaluronic acid droplets in a pre-warmed Falcon 3002 Petri dish, and the CCs were mechanically dissociated. Due to hyaluronidase’s strong digestion ability, it was unsuitable for excessive digestion, generally not more than 1 min, to reduce damage to CCs and oocytes. Rapid transfer of COC to be separated into pre-prepared ICSI operating fluid, repeat gently a few more times until completely separated. Then, the naked oocyte removed was quickly transferred to the operating dish for marking, and the maturation stage was assessed and recorded by observing the nucleus of the oocyte. Oocytes with an extruded polar body were deemed MII oocytes, oocytes with an intact germinal vesicle were deemed GV oocytes, and oocytes without an observable germinal vesicle or an extruded polar body was deemed MI oocytes. A total of 42 oocytes (GV:22, MI:11, MII:9) and 97 CCs (GV:35, MI:30, MII:32; randomly selected 10 cells from individually COC per CC samples to avoid bias due to the different number of CCs in each follicle) were getting. After cleaning, each sample was placed in a separate EP tube that had been added to the RNA lysate for lysis (or stored in a -80℃ refrigerator).

### RNA-Seq library construction and sequencing

Single-cell was lysed by RNA lysates, and RNA was released and converted into cDNA using the Smart RNA-seq2 protocol as previously described [46], the sequencing technology widely used for its high sensitivity and specificity. Briefly, Superscript II reverses transcriptase, and oligo-dT primers are used to specifically reverse RNA with the poly (A) tail. Once reversed to the 5′ ends of the RNA molecule, reverse transcriptase adds a C base to the 3′ ends of cDNA. The template-switching oligonucleotide modified with Locked Nucleic Acid is then used to pair with C, and the second strand is synthesized. After template conversion, the resulting cDNA contains the complete sequence of the mRNA 5 end and the anchor sequence for the synthesis of the second cDNA strand, which is then amplified. The library was amplified to make a DNA nanoball (DNB) which had more than 300 copies of one molecular. The DNBs were loaded into the patterned nanoarray, and single end 50 bases reads were generated in the way sequenced by combinatorial Probe-Anchor Synthesis(cPAS) (BGI, Shenzhen).

### Post-sequencing quality control

1. Raw reads were obtained by transcriptome sequencing. Then, FASTP (V0.20.0) was used to remove the data including low-quality reads (reads with too many low-quality bases or unknown base N content) and joint contamination (too short, imported fragments led to the detection of joint sequences on both sides), and the filtered data, namely clean reads, were obtained. FASTP was used to evaluate raw data and clean data before and after filtration, including base quality, sequence length, base ratio, GC content, repeat sequence, etc.
2. STAR (V2.7.3a) was used to compare reads with the human reference genome (without masking repeats) of GRCh38, and the mapping rate of sequencing data was counted. Cells were quality-filtered based on two criteria, the number of expressed genes per cell was used to identify one outlier cell with less than 5000 expressed genes, and the outlier cells were identified using PCA.
3. Calculate the expression amount of the BAM file after Mapping to get the count of each gene. Normalization of reads counts on Transcripts: Calculation and normalization of the Transcripts Per Million (TPM) using String Tie (V2.0.4). Differential gene analysis using DESeq2 R package, including the construction of DDS matrix, standardization, and differential analysis.) To improve the efficiency of identifying DEGs, only informative genes with at least 20% of samples having at least 10 reads were retained. After filtering out cells and genes, the Bayesian-based approach SCDE (v1.99.4) (that model's technical noise and biological variability were used to screen for DEGs (*p* < 0.5 and |log_2_(fold change) |≥ 2) between the different stages. Gene ontology enrichment analysis was based on Fisher’s exact test, with all human protein-coding genes used as the background set.

According to the results of differential gene analysis, the R software packages hclust and heatmap were used to cluster analysis of Pearson correlation among the samples, and the visualized heatmap and unsupervised principal component analysis (PCA) diagram were used for transcriptome differences of these samples.

### Bioinformatics

According to the results of differential gene analysis, R software package hclust, and Pheatmap were used for clustering analysis of Pearson correlation between samples, and visual heatmap and principal component analysis diagram were output.

Differential gene function enrichment analysis and data visualization: R software package ClusterProfile: GO enrichment analysis of differential genes. The list of biological process (BP), molecular function (MF), and cellular component (CC) of differential genes were output respectively. Metascatpe online database for gene function analysis:https://metascape.org/gp/index.html#/main/step1 The website integrates GO Term, KEGG, Reactome, and BioCarta, as well as MSigDB multiple pathways. The above two databases enriched the genes with significant differences and drew corresponding annotation maps.

### Supplementary Information

**Additional file 1: Supplementary Table 1. **Clinical characteristics and outcome of patients. **Supplementary Table 2.** Age of patients at different maturation stages. **Supplementary Table 3.** Sample size data of two groups categorized by age at different maturation stages. **Supplementary Figure 1.** DE analysis between oocytes and CCs. **Supplementary Figure 2.** Clustering profiles of all CCs samples. **Supplementary Figure 3.** qPCR results of seven DEGs involved in cholesterol metabolism and fatty acid metabolism. **Supplementary Figure 4.** GO enrichment of differentially up-regulated genes in age ≥ 40 years patients’ MI oocytes.

## Data Availability

The raw data have been deposited to CNCB-NGDC Genome Sequence Archive for Human (accession number: HRA002098; https://ngdc.cncb.ac.cn/gsa-human/browse/HRA002098).
