# Supplementary material for: Transcriptomic profiles reveal the characteristics of oocytes and cumulus cells at GV, MI, and MII in follicles before ovulation
Source: J Ovarian Res. 2023 Nov 22;16:225. doi: 10.1186/s13048-023-01291-2 (PMC10664256; doi:10.1186/s13048-023-01291-2)
Supplement: Supplementary file 1 — Additional file 1: Supplementary Table 1. Clinical characteristics and outcome of patients. Supplementary Table 2. Age of patients at different maturation stages. Supplementary Table 3. Sample size data of two groups categorized by age at different maturation stages. Supplementary Figure 1. DE analysis between oocytes and CCs. Supplementary Figure 2. Clustering profiles of all CCs samples. Supplementary Figure 3. qPCR results of seven DEGs involved in cholesterol metabolism and fatty acid metabolism. Supplementary Figure 4. GO enrichment of differentially up-regulated genes in age ≥ 40 years patients’ MI oocytes. [file 13048_2023_1291_MOESM1_ESM.docx]

Supplementary Table 1. Clinical characteristics and outcome of patients.

|  | Mean±SEM |
| --- | --- |
| Age(years) | 35.1±5.8 |
| BMI (kg/m^2^)  FSH (mIU/ml)  LH (mIU/ml)  AMH (mIU/ml) | 21.8±2.5  7.6±3.5  4.7±2.6  2.8±1.9 |
| AFC (antral follicle) | 15.9±7.5 |
| Oocytes retrieved | 8.7±2.7 |

Supplementary Table 2. Age of patients at different maturation stages.

| Stages | Age(years)Mean±SEM |
| --- | --- |
| GV (oocytes) | 33.5±7.0 |
| MI（oocytes） | 33.4±5.7 |
| MII（oocytes） | 27.7±3.3 |
| GV (cumulus cells) | 34.6±6.2 |
| MI（cumulus cells） | 34.2±5.0 |
| MII（cumulus cells） | 36.5±5.7 |
| Total | 35.1±5.8 |

Supplementary Table 3. Sample size data of two groups categorized by age at different maturation stages.

| Stages | Age ≤30 years (n) | Age ≥40 years (n) |
| --- | --- | --- |
| GV (oocytes) | 10 | 5 |
| MI（oocytes）  MII（oocytes）  GV (cumulus cells)  MI（cumulus cells） | 5  7  10  10 | 2  0  7  5 |
| MII（cumulus cells） | 8 | 13 |

Supplementary Figure 1. DE analysis between oocytes and CCs.


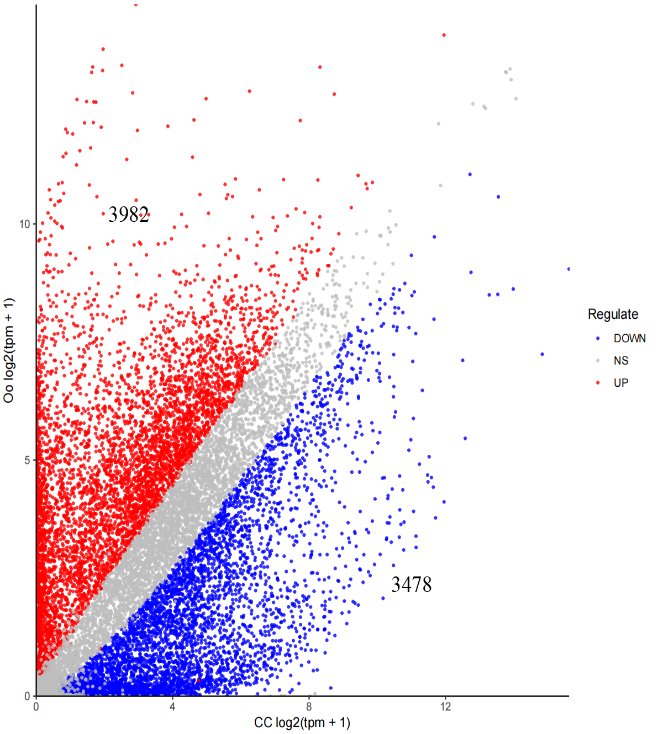


Supplementary Figure 2. Clustering profiles of all CCs samples


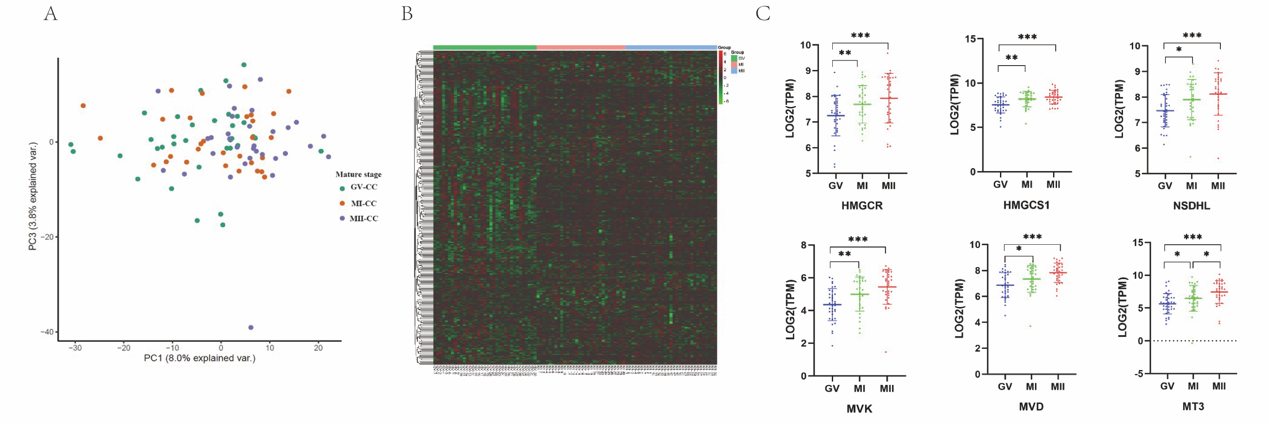


(A) PCA showed that the clustering characteristics of all CCs samples at the transcriptome level were not prominent.

(B) Heatmap analysis of differential genes in CC.

(C) Expression pattern diagram of some key genes involved in de novo cholesterol synthesis in three periods. HMGCR (3-hydroxy-3-methylglutaryl coenzyme A reductase); HMGCS1 (3-hydroxy-3-methylglutaryl CoA synthase 1); NSDHL (NAD(P) dependent steroid dehydrogenase-like); MVK (Mevalonate kinase); MVD (pyrophosphomevalonate decarboxylase); MT3 (metallothionein 3). GV, blue; MI, green; MII, red

Supplementary Figure 3. qPCR results of seven DEGs involved in cholesterol metabolism and fatty acid metabolism


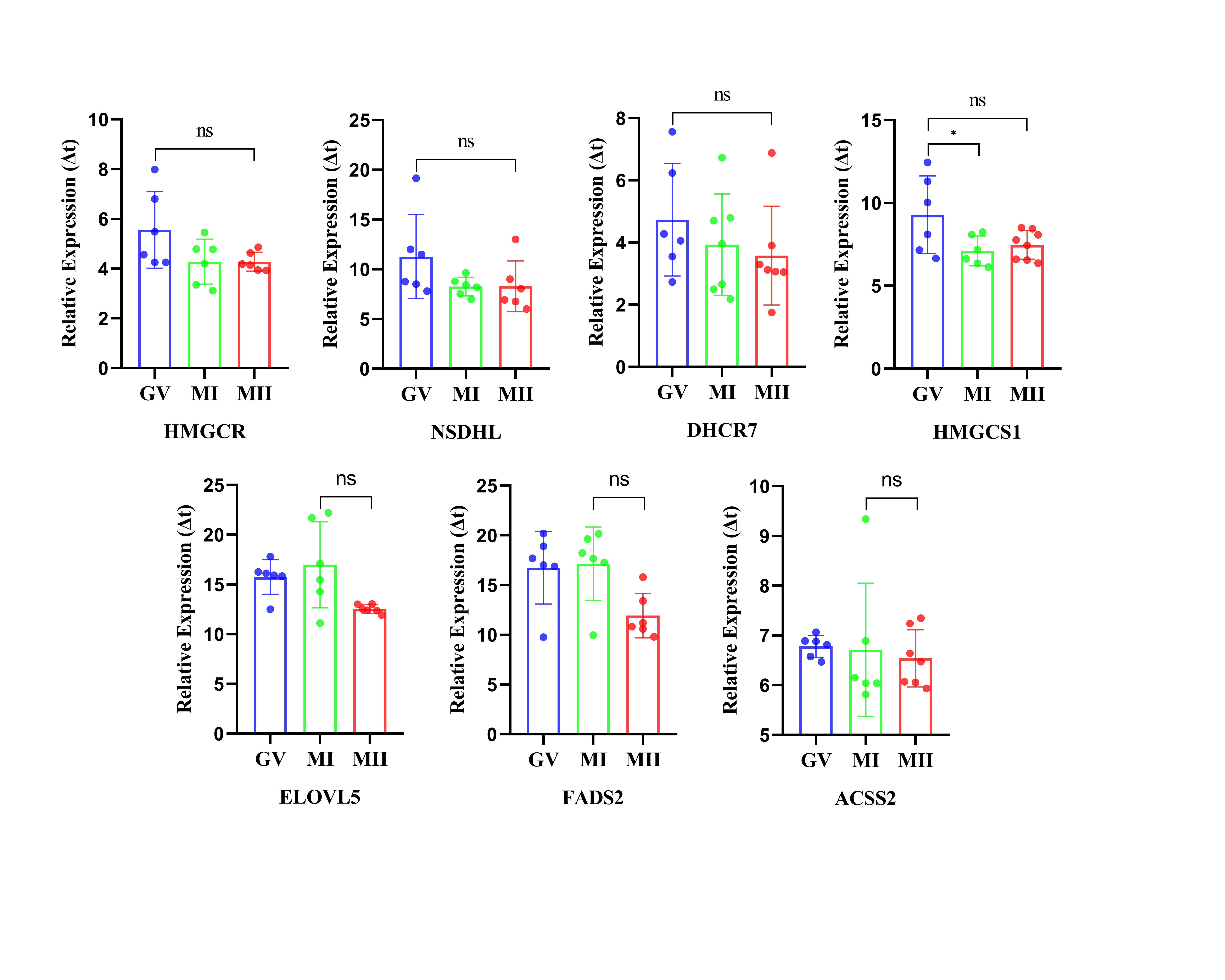


Supplementary Figure 4. GO enrichment of differentially up-regulated genes in age≥40 years patients’ MI oocytes


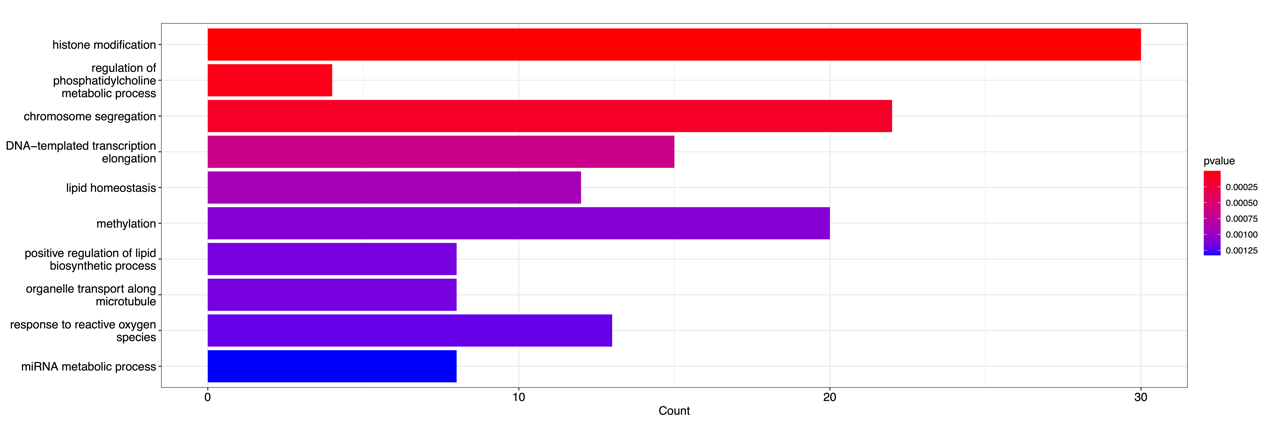


GO enrichment of differentially up-regulated genes in age≥40 years patients’ MI oocytes. The sizes of the points indicate the number of genes enriched in the pathway. The X-axis indicates significance.
